# Sex-specific induction of CYP6 cytochrome P450 genes in cadmium and lead tolerant *Anopheles gambiae*

**DOI:** 10.1186/1475-2875-12-97

**Published:** 2013-03-15

**Authors:** Fauzia K Musasia, Alfred O Isaac, Daniel K Masiga, Irene A Omedo, Ramadhan Mwakubambanya, Richard Ochieng, Paul O Mireji

**Affiliations:** 1 Department of Biochemistry and Molecular Biology, Egerton University, P.O. Box 536, Njoro, Kenya; 2 Molecular Biology and Bioinformatics Unit, International Centre of Insect Physiology and Ecology, P.O. Box 30772–00100, Nairobi, Kenya; 3 Centre for Biotechnology and Bioinformatics, University of Nairobi, P.O. Box 29053–00625, Nairobi, Kenya

**Keywords:** *Anopheles gambiae*, Cadmium, Lead, Cytochrome p450

## Abstract

**Background:**

*Anopheles gambiae,* one of the main Afro-tropical mosquito vector of malaria, has adapted to heavy metals in its natural habitat, and developed resistance to most conventional insecticides. Investigations were conducted to establish an association between tolerance to cadmium or lead-heavy metals, and expression of specific genes for cytochrome p450 enzymes associated with pyrethroid resistance in the mosquito.

**Methods:**

Juvenile aquatic stages of the mosquito were selected for tolerance to cadmiun or lead through chronic exposure of the stages to maximum acceptable toxicant concentrations (MATCs) of the metals. Using real-time quantitative polymerase chain reaction (qPCR), three replicates each of male or female cadmium or lead-tolerant individuals and relevant controls were separately screened for expression of *CYP6M2*, *CYP6P3* and *CYP6Z1* genes. The variance in expression levels of the genes amongst the treatments was compared by ANOVA statistical tool.

**Results:**

Expressions of all the genes were significantly lower (P <0.05) in females than in males. Within gender, there 1.3 - 2.3 or 3.1-4.2-fold reduction in expression of the genes in cadmium or lead selected than respective control populations. Expression of all the classes of gene was elevated in cadmium selected female populations relative to their respective controls.

**Conclusion:**

These findings suggest that tolerance to cadmium or lead in the mosquito can influence response in cytochrome p450 genes associated with metabolism of pyrethroids in the mosquito in a sex-specific manner. This can, in turn, affect sensitivity of the mosquito to pyrethroids and other xenobiotics associated with these genes, with potential implications in mosquito vector control operations.

## Background

*Anopheles gambiae*, one of the main African vector of malaria, is expanding its ecological niche into urban areas where larvae of the mosquito have often been observed proliferating in polluted aquatic habitats [1,2]. The pollutants include domestic and/or industrial sewage [1,3], and heavy metals in excess of natural loads [2]. The mosquito has also developed resistance to conventional insecticides, including pyrethroids, following extensive application of the insecticides in control programmes of the vector in rural and urban Africa [4,5]. The adaptation to the metals and resistance to the insecticides has in turn facilitated colonization by the mosquitoes of polluted habitats that are potentially unfavourable to susceptible predators and competitors of the mosquitoes [2,6-9]. Resistance has been facilitated by metabolism of the pyrethroids by *CYP6M2*, *CYP6P3* and *CYP6Z1* genes of CYP6 family of cytochrome p450 enzymes in the mosquito [10-12]. Evolutionary pressures have also driven catalytic plasticity in these enzymes, facilitating rapid expansion and diversification of functions of the genes, to potentially handle additional xenobiotics, including heavy metals [13]. The roles of these genes in mediating resistance to pyrethroids in *Anopheles gambiae* mosquito tolerant to cadmium, copper or lead heavy metal have not been elucidated.

However, tolerance to heavy metals in the mosquito has been associated with loss in biological fitness of the mosquito with potential implication in their ecological performance [14-16]. Therefore, understanding the influence of heavy metal tolerance on CYP6 gene expression responses in the mosquito can potentially elucidate impact of adaptation of the mosquitoes to the metals in nature and on the susceptibility of the mosquitoes to the pyrethroid insecticides routinely used in malaria control interventions against the vector [17]. In this study, the effect of tolerance in *An. gambiae* s.s. to cadmium and lead-heavy metals on expression of the *CYP6M2*, *CYP6P3* and *CYP6Z1* genes of CYP6 family of cytochrome p450 in the species was assessed.

## Methods

### Selection for tolerance to cadmium or lead in *Anopheles gambiae s.s*

*Anopheles gambiae s. s.* mosquitoes were obtained from a colony kept by the Animal Rearing and Containment Unit (ARCU) of International Centre of Insect Physiology and Ecology (ICIPE), Nairobi, Kenya. This colony was originally collected from Mbita field station (00.025’S, 34.013’E), Homa Bay County, Kenya in December 2000. Cadmium or lead-heavy, metal-tolerant, and control strains of the mosquito were separately generated and maintained through exposures of successive generations of the mosquito to chronic concentrations of the metals in three independent replicates for each strain and treatment since 2006 as described in [15]. Briefly, larval stages of *An. gambiae s. s.* were selected for tolerance to cadmium or lead through chronic exposures to empirically determined maximum acceptable toxicant concentration (MATCs) of the metals. At the time of this work, the colony had been selected for tolerance to respective heavy metals through at least 143 filial generations. Overall, maintenance of the colony followed standard operating procedure for rearing *Anopheles*, where female mosquitoes were blood-fed on anaesthetized mice, and larvae on pulverized Tetramin fish food (Tetra GmbH, Melle, Germany) [18]. Approval for feeding mosquitoes on mice was obtained from the Kenya National Ethical Review Board (protocol number KEMRI/RES/7/3/1), and the protocol reviewed by the KEMRI IACUC. Cadmium and lead used in this study were applied as cadmium chloride (CdCl_2_) 99.99% pure (Fisher Scientific LLC, Fair Lawn, NJ, USA) and lead II nitrate [Pb (NO_3_)_2_] 99.5% pure analytical salts (Prolabo, Fontenay-sous-Bois, France).

### Extraction of ribonucleic acid from adult *Anopheles gambiae s.s*

Total ribonucleic acid (RNA) was separately isolated from pools of the three independent replicates, each pool consisting of five three-day-old male or female adult mosquitoes of cadmium or lead-tolerant strain, or their respective untreated controls. RNA was isolated using TRIzol reagent (Invitrogen, Carlsbad, USA) following the manufacturer’s protocol. The resultant RNA from each pool was separately suspended in 12 μL of nuclease-free water and stored at −80°C until required. Integrity of extracted RNA was validated by electrophoresis in 0.3% agarose (Sigma-Aldrich hemie, Gmbh, Steinheim, Germany) RNA denaturing gel in 1.4% sodium phosphate, with 1 mg/mL ethidium bromide staining for visualization. The yields and quality of RNA were determined spectroscopically [19], and the RNA subsequently used for cDNA synthesis.

### Reverse transcriptions of genes for cytochrome p450

Two-steps reverse transcriptions of RNA, and Dnase treatment to remove potential genomic DNA carryover/contaminants were conducted using RevertAid™ H Minus First Strand Synthesis Kit (Fermentas, Lithuania) following manufacturer’s instructions. The DNase treatment reaction consisted of 1 μg RNA extract, 1 μL 10X reaction buffer with MgCl_2_, 1 uL of RNase-free DNase I and nuclease-free water in total reaction volumes of 10 μL. The reactions were incubated at 37°C for 30 min, followed by the addition of 1 μL 50mM EDTA, and a further incubation for 10 min at 65°C. Resultant RNA from each strain was separately denatured by incubation at 65°C for 5 min in 12 μL reactions mix consisting of 1 μg RNA, 1 μL oligo (dT)_18_ primer and 1 μL nuclease-free water. Subsequent cDNA syntheses were separately conducted by the addition of 4 uL5X reaction buffer, 20 uL RiboLock RNase inhibitor, 2 μL 10 mM dNTP Mix and 200uL RevertAid H Minus M-MuLV reverse transcriptase in total reaction volumes of 20 μL. The reactions were incubated at 45°C for 1 hour, and reverse trancriptase subsequently inactivated at 70°C for 5 min. The cDNA generated was stored at −20°C.

### Real-time qRT-PCR of genes for cytochrome P450

Expression profiles of the *CYP6M2*, *CYP6P3* and *CYP6Z1* genes for cytochrome P450 enzymes in the mosquito strains tolerant to cadmium or lead, and their respective controls, were separately assessed. These genes have been associated with resistance to pyrethroids [10-12], and are potentially responsive to xenobiotics such as heavy metals [13]. Reaction volumes of 15 μL for each strain consisting of 3 μg cDNA template were amplified in three independent replicates with 7.5 μL of Fast SYBR® Green Master Mix (Applied Biosystems, Carlsbad, CA, USA) in the presence of 0.5 picomoles of specific primers for *CYP6M2*, *CYP6P3* and *CYP6Z1* and Ribosomal protein L19 (*RPL19*) internal control (Table 1). The reactions were carried out in a real-time qPCR machine (Strategene MX3005P, Aligent Technologies, CA, USA) according to the manufacturer’s instructions. The thermo-cycling conditions involved an initial step of 95°C for 30 sec, 40 cycles of 95°C for 3 sec, 60°C for 30 sec, followed by one cycle of 95°C for 30 sec, 60°C for 1 min and 95°C for 30 sec for all the genes.

**Table 1:** **Cytochrome p450 and*RPL19*-specific primers for qPCR**

**Gene**	**Accession number**	**Sequence (5'-3')**	**Expected size of product (bp)**
*CYP6M2*	AGAP008212-RA	^F^ 5' TTCGTCGACTCTCCTCACCT 3'	299
		^R^ 5' GAAATGTACCGGGACTGGTG 3'	
*CYP6P3*	AGAP002865-RA	^F^5' AGCTAATTAACGCGGTGCTG 3'	121
		^R^ 5' AAGTGTGGATTCGGAGCGTA 3'	
*CYP6Z1*	AF487535	^F^ 5' TTACATTCACACTGCACGAG 3'	146
		^R^ 5' CTTCACGCACAAATCCAGAT 3'	
*RPL19*	XM_001864829.1	^F^ 5' CCAACTCGCGACAAAACATTC 3'	75
		^R^ 5' ACCGGCTTCTTGATGATCAGA 3'	

*CYP6M2*, *CYP6P3* are *CYP6Z1*are classes M2, P3 and Z1 respectively in family 6 of cytochrome p450 genes; *RPL19* is ribosomal protein L19 internal control; ^F^ or ^R^ superscript are forward or reverse primers respectively.

### Data analysis

Relative expressions of *CYP6M2*, *CYP6P3* and *CYP6Z1* genes were calculated using GenEx 5.3.6 [20]. Briefly, relative quantities of expression of *CYP6M2*, *CYP6P3*, *CYP6Z1* and *RPL19* genes were determined in relation to a control sample in the female condition, being the sample with the lowest expression in all gene categories. The expressions of *CYP6M2*, *CYP6P3* and *CYP6Z1* were subsequently normalized with *RPL19* reference gene using the geNorm function in GenEx. For the qRT-PCR statistical analysis, the expression data were log transformed (Log10) to normalize the data and homogenize the variance prior to two-way analysis of variance where effect of gene, sex and interaction were evaluated. Means were separated by Tukey’s honestly significant difference (HSD) test and detransformed for reporting. All data analyses were implemented using R-Development-Core-Team [21].

## Result and Discussion

Relative expression profiles of *CYP6M2*, *CYP6P3* and *CYP6Z1* genes of CYP6 family of cytochrome p450 genes in cadmium and lead-tolerant *An. gambiae s. s*, and their respective controls are summarized in Table 2. Expressions were significantly lower in females than males in *CYP6M2* (F_1,17_ = 21.35, P <0.001 ), *CYP6P3 (*F_1,17_ = 7.35, P = 0.019*)* and *CYP6Z1* (F_1,17_ =11.54, P= 0.005) genes. Within gender, there was between 3.1 and 4.2-fold reduction in expression of the genes in lead-selected than control among male populations. Similarly, there was between 1.2 and 2.2-fold reduction in expression of the genes in cadmium-selected than respective male control populations. The reductions were not statistically significant. In both cases, most pronounced reductions were observed in expression of *CYP6P3.* However, the expression of all the classes of genes was marginally elevated in cadmium-selected female populations relative to their respective controls. Expressions of all the genes of CYP6 family of cytochrome450 genes assessed in this study were generally depressed by cadmium or lead tolerance in the mosquito in a sex-dependent manner.

**Table 2:** **Relative mean (± SE) expression levels of various cytochrome p450 genes in heavy metal-tolerant adult*Anopheles gambiae*s.s mosquito**

**Gender**	**Treatment**	***CYP6M2***	***CYP6P3***	***CYP6Z1***
Males	Cont	48.386 ± 15.082	25.894 ± 12.015	28.220 ± 11.883
	Cd	38.052 ± 8.370	11.196 ± 3.280	18.287 ± 6.771
	Pb	15.820 ± 4.858	6.110 ± 1.663	8.999 ± 1.959
Females	Cont	6.347 ± 4.241	5.853 ± 2.456	5.737 ± 3.351
	Cd	9.694 ± 3.826	6.006 ± 1.727	6.998 ± 1.535
	Pb	5.145 ± 1.235	3.831 ± 0.800	4.678 ± 1.267

*CYP6M2*, *CYP6P3* are *CYP6Z1*are classes M2, P3 and Z1 respectively in family 6 of cytochrome p450 genes. Cont, Cd and Pb represent control, cadmium and lead-tolerant populations of *Anopheles gambiae s.s.* respectively.

Factors underlying the observed differential responses to the metals in the mosquito are not apparent. Although xenobiotics-associated induction of cytochrome p450 genes in *An. gambiae* inhabiting polluted water bodies has putatively been linked to induction of resistance to pyrethroids in the mosquito, [1,3,22], The findings point to the contrary. Heavy metal exposure down-regulated expression of cytochrome p450 gene [23,24]. Published data suggest that the depressed expression observed in this study could be linked to cadmium induction of haem oxygenase associated with haem degradation [23] or lead inhibition of gamma aminolevilinic acid dehydratase associated with synthesis of haem [25]. Either case would deplete haem, a major component of the CYP6 family of cytochrome p450 gene, precipitating observed reduced expression of the gene. Similarly, sex-specific responses in cadmium-tolerant populations may be linked to female-specific, cadmium-binding protein in the mosquitoes which might have reduced bioavailability of cadmium to cytochrome p450, and subsequently reduced cadmium induced heme-degradation and hence depletion of CYP6 family of cytochrome 450 gene [23]. Further bioassay studies on metal tolerant populations will shed light on influence of the observed genes expression dynamics on susceptibility of the mosquito to classes of insecticides, including pyrethroids, metabolised by CYP6 family of cytochrome 450 enzyme.

## Conclusion

Tolerance to cadmium or lead in *An. gambiae* s.s significantly influences expression of *CYP6M2*, *CYP6P3* and *CYP6Z1* genes of CYP6 family of cytochrome p450 gene in a metal and sex-specific manner. These changes in gene expression profiles can potentially affect susceptibility of the mosquito to pyrethroid insecticides, and influence vector control efforts.

## Competing interests

The authors declare that they have no competing interests

## Authors’ contributions

FKM, DKM and POM conceived the study and participated in the implementation, data analysis, interpretation and manuscript preparation. IAO, AOI and RM guided the study from the conception to manuscript finalization. RO participated in the design of the study. All authors read and approved the final manuscript.
